# Probing rapid carbon fixation in fast-growing seaweed *Ulva meridionalis* using stable isotope ^13^C-labelling

**DOI:** 10.1038/s41598-020-77237-1

**Published:** 2020-11-23

**Authors:** Shuntaro Tsubaki, Hiroshi Nishimura, Tomoya Imai, Ayumu Onda, Masanori Hiraoka

**Affiliations:** 1 School of Materials and Chemical Technology, Tokyo Institute of Technology, E4-3, 2-12-1, Ookayama, Meguro-ku, Tokyo, 152-8552 Japan; 2 Research Institute for Sustainable Humanosphere, Kyoto University, Gokasho, Uji, 611-0011 Japan; 3 Research Laboratory of Hydrothermal Chemistry, Faculty of Science, Kochi University, 2-17-47 Asakurahonmachi, Kochi, 780-8073 Japan; 4 Usa Marine Biological Institute, Kochi University, Inoshiri, Usa, Tosa, Kochi 781-1164 Japan

**Keywords:** Carbohydrates, Biofuels, Photosynthesis

## Abstract

The high growth rate of *Ulva* seaweeds makes it a potential algal biomass resource. In particular, *Ulva meridionalis* grows up to fourfold a day. Here, we demonstrated strong carbon fixation by *U. meridionalis* using ^13^C stable isotope labelling and traced the ^13^C flux through sugar metabolites with isotope-ratio mass spectrometry (IR-MS), Fourier transform ion cyclotron resonance mass spectrometry (FT-ICR-MS), ^13^C-nuclear magnetic resonance spectrometry (^13^C-NMR), and gas chromatography-mass spectrometry (GC–MS). *U. meridionalis* was first cultured in ^13^C-labelled enriched artificial seawater for 0–12 h, and the algae were collected every 4 h. *U. meridionalis* grew 1.8-fold (dry weight), and the ^13^C ratio reached 40% in 12 h, whereas ^13^C incorporation hardly occurred under darkness. At the beginning of the light period, ^13^C was incorporated into nucleic diphosphate (NDP) sugars in 4 h, and ^13^C labelled peaks were identified using FT-ICR-MS spectra. Using semiquantitative ^13^C-NMR measurements and GC–MS, ^13^C was detected in starch and matrix polysaccharides after the formation of NDP sugars. Moreover, the 14:10 light:dark regime resulted into 85% of ^13^C labelling was achieved after 72 h of cultivation. The rapid ^13^C uptake by *U. meridionalis* shows its strong carbon fixation capacity as a promising seaweed biomass feedstock.

## Introduction

Green tides of *Ulva* seaweeds are increasing with the eutrophication of the sea and have become a major environmental problem^1^. Rapid and vast *Ulva* growth disturbs the ecological system at the seashore. The growth dynamics and mechanisms of fast growth in *Ulva* have been studied to mitigate *Ulva* green tides^2–4^. At the same time, *Ulva* is an expected biomass source that can capture CO_2_ and convert it into renewable biochemicals^5^. Filamentous *Ulva meridionalis* is a potential seaweed species that exhibits the fastest growth rate ever found in multicellular autotrophic plants^6,7^. *U. prolifera*, one of the most widely known edible *Ulva* species, doubles in a day at the optimised culturing conditions; in contrast, *U. meridionalis* grows over fourfold in a day. In general, many *Ulva* grow from winter to spring, whereas *U. meridionalis* grows in summer. *U. meridionalis* has a high rate of photosynthetic carbon fixation, a high frequency of cell proliferation, and rapid formation of algae bodies consisting of cell walls in high-temperature environments. The accumulated cell and cell wall components are important biochemical resources that include polysaccharides and rare sugars^5^, which are produced by *Ulva* through extraction and hydrolysis processes^8–10^. Land cultivation of *Ulva* has been extensively studied for stable seaweed production for both food and biomass applications^11^. The “germling cluster method” allows mass production of *Ulva* by clustering the algae bodies and cultivating them in high density floating *Ulva* clusters in a land tank. This method provides efficient exposure for *Ulva* growth by three-dimensionally stirring the clusters. *Ulva* are easily collected by a coarse filtration, which is much easier than harvesting microalgae biomass that requires flocculation and centrifugation. This efficient cultivation system enhances *Ulva* growth and captures more CO_2_.

*Ulva* suffers various environmental stresses such as frequent submergence into seawater and exposure to air by tides, high light intensity, and dryness at the intertidal zone. However, their photosynthetic systems are highly adapted to severe environments^12^. At the same time, *Ulva* are widely distributed worldwide and are a model macroalgae that are used for studying photosynthesis mechanisms^13^. Most macroalgae utilise bicarbonate ions (HCO_3_^−^) as a carbon source because it is a major species of CO_2_ that occurs at the pH and salt concentration levels of seawater^14^. Carbonic anhydrase shifts the equilibrium of HCO_3_^−^ and CO_2_ to enhance CO_2_ uptake^15^. Several macroalgae, including *Ulva*, have a proton pump system that enhances the HCO_3_^−^ uptake. Moreover, *Ulva* is equipped with a carbon concentrating mechanism (CCM). Beer et al. reported that the introduction of ^14^C pulses into *U. fasciata* under light immediately produces ^14^C labelled glycerate 3-phosphate with more than 90% of molecules labelled, and it was further transferred to other photosynthesis products such as sugars^16^. Since photorespiration is suppressed in *Ulva*, they concluded that *Ulva* have a carbon concentrating system in their chloroplasts along with their efficient HCO_3_^−^ uptake mechanism^17,18^. The CCM maintains the CO_2_ in cells at a sufficiently high level to saturate carboxylation by Rubisco in the Calvin cycle^17^. The CCM was also found in *U. prolifera*, which caused huge green tides in the Yellow Sea^19^. *Ulva* also exhibits high tolerance to strong light by non-photochemical quenching. Efficient photosynthesis can be maintained under strong light by protecting photosystems I and II from excess electrons (e^−^) by trapping them in xanthophyll^20,21^.

Various polysaccharides such as storage, cell wall, and matrix polysaccharides are formed through biosynthetic pathways after carbon fixation in the Calvin cycle. *Ulva* bodies consist of cellulose (~ 10%), matrix polysaccharides (~ 30%), and starch (~ 10%) along with proteins (~ 20%)^8^. *Ulva* seaweeds are characterised by matrix polysaccharides (ulvan) that are heteropolysaccharides consisting of rhamnose (Rha), glucuronic acid (GlcA), iduronic acid (IdoA), and sulfates^22–24^. In some cases, mannose (Man), galactose (Gal), and arabinose (Ara) are found in ulvan. Various fine structures were reported including: (1) Type-A ulvanbiouronic 3-sulfate (A_3S_), β-d-GlcA (1 → 4)-l-Rha 3S → 4)β-d-GlcA (1 → 4)-α-l-Rha 3S (1 → ; (2) Type B ulvanbiouronic 3-sulfate (B_3S_), α-l-IdoA (1 → 4) α-l-Rha 3S → 4) α-l-IdoA (1 → 4)-α-l-Rha 3S (1 → ; (3) ulvanbiose 3-sulfate (U_3S_), → 4) β-d-Xyl (1 → 4) α-l-Rha 3S (1 → ; (4) ulvanobiose 2′,3-disulfate (U_2_′_s,3s_), → 4) β-d-Xyl 2S (1 → 4) α-l-Rha 3S (1 →^23,24^. Biosynthesis of ulvan occurs from nucleic diphosphate (NDP) sugar, similar to pectin biosynthesis in higher plants^23^. d-fructose-6-P supplied from photosynthesis and glucogenesis is converted to uridine diphosphate glucose (UDP-Glc) through several steps. The corresponding NDP sugars to each monosaccharide are produced through the interconversion of UDP-Glc. For instance, UDP-GlcA is converted from UDP-Glc or inositol and further converts to UDP-Xyl. Rha originates from UDP-L-Rha or dTDP-L-Rha, which are converted from UDP-Glc. Ulvan is synthesised by glycosyltransferase from the above-mentioned NDP sugars in the Golgi apparatus. Polysaccharides are then transported to the apoplast, and further assembling and modifications occur. However, the biosynthetic pathway of ulvan is yet not well understood although it is involved in the rapid growth of *Ulva*.

Isotope labelling has been used to analyse the kinetics of carbon uptake by photosynthesis and its transfer into metabolites. Percival et al. used the radioisotope ^14^C to study carbon fixation of *U. lactuca* and its transfer into sugar metabolites^25^. They reported that ^14^C incorporation in 80% aqueous ethanol extract or hot water extract was detected after culturing in a ^14^C medium containing NaH^14^CO_3_ for 10 min. Incorporation of ^14^C into starch occurred earlier than ulvan. After the hydrolysis of ulvan, the radioactivity of Rha was higher than that of GlcA, indicating that Rha deposition occurs earlier than GlcA deposition. Recent advances in analytical instruments such as mass spectrometry (MS) and nuclear magnetic resonance (NMR) allow for a more comprehensive analysis of fluxes of metabolites and material exchanges between organisms by stable isotope labelling^26–28^. Natural ^13^C distribution is only 1.07%, but concentrating ^13^C makes structural analysis of metabolites by ^13^C-NMR spectroscopy easier. High performance liquid chromatography electron spray ionization mass spectrometry (HPLC ESI–MS) and GC–MS analysis of ^13^C-enriched metabolites enable tracing of the ^13^C flux of each component. Further, FT-ICR-MS provides an accurate mass of metabolites that allows tracing of the metabolic foot print^29^.

This study demonstrated the behaviour of rapid carbon fixation in *U. meridionalis* and its transfer to polysaccharides through NDP sugars to understand the physiological mechanisms of their extremely high growth rate. *U. meridionalis* was cultured in enriched artificial seawater (EASW)^30–32^ medium with ^13^C-NaHCO_3_ as the sole carbon source, and the temporal ^13^C transfer to NDP sugars and polysaccharides (ulvan and starch) was analysed. Incorporation of ^13^C into NDP sugars was analysed using FT-ICR-MS. Semiquantitative ^13^C-NMR by inverse-gated-decoupling was used to observe ^13^C incorporation into polysaccharides. GS-MS was used to trace ^13^C uptake in monosaccharide moieties in polysaccharides after their acid hydrolysis. Finally, the flux of carbon fixation and its transfer into the cell wall polysaccharides was discussed in conjunction with the rapid growth rate of *U. meridionalis*.

## Results

### ^13^C uptake by *U. meridionalis*

**Figure 1:**
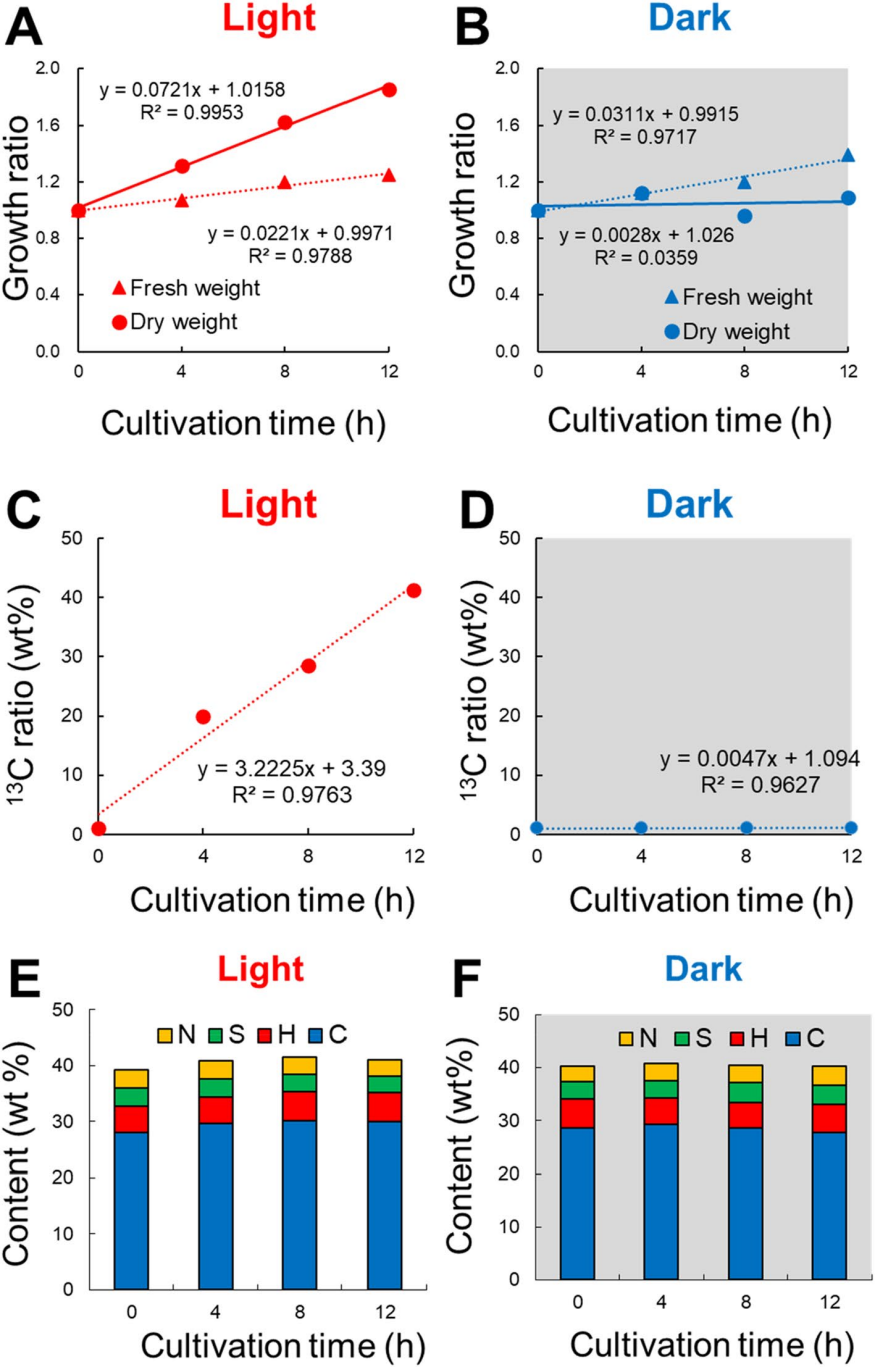
^13^C uptake by *Ulva meridionalis* under light and dark conditions. Fresh and dry growth rate under (**A**) light and (**B**) dark. ^13^C uptake under (**C**) light and (**D**) in the dark. The elemental composition of ^13^C-labelled *U. meridionalis* under (**E**) light and (**F**) dark.

Figure 1A,B shows the changes in fresh weight and dry weight over time in light and dark conditions. The fresh weight shows linear increases over time, and it doubled in 24 h. *U. meridionalis* grew up to about 3.5-fold in a day at the same light and temperature condition (12:12 light:dark regime, 25 °C)^6^. In the present study, the growth rate of *U. meridionalis* was lower because closed flasks were used for its cultivation, without aeration, to prevent an exchange of ^13^C with ^12^C in the atmosphere. Therefore, diffusion of oxygen, which is an inhibitor of the carboxylase activity of Rubisco, might not be sufficient, resulting in a lower growth rate than the ideal one. Moreover, ^13^C uptake can be slower than ^12^C uptake because of its larger mass. The dry weight of *U. meridionalis* increased by 1.8 times, and the amount of oxygen generated reached 49 mL after 12 h in the light period (Supplementary Fig. S1). In the dark period, the fresh weight increased linearly at the same rate as that under light, but the dry weight did not change. That is, photosynthetic metabolites accumulated in the light period to increase their dry weight. Meanwhile, water content increased during the dark period.

After powdering the dried sample, the amount of incorporated ^13^C was measured by IR-MS. The ^13^C uptake in the light period was 41.2%, which was consistent with a doubling of dry weight under light (Fig. 1C). On the other hand, ^13^C uptake was hardly observed in the dark period, indicating that the carbon fixation of *U. meridionalis* occurs in a light-dependent manner (Fig. 1D). Then, the elemental composition was investigated by CHNS analysis. The amounts of C (27.8% to 30.2%) and H (4.7% to 5.5%) reflected entire organic compounds, including sugar chains and proteins (Fig. 1E,F). The amount of N (3.0% to 3.9%) was mainly attributed to protein and that of S (2.9% to 3.9%) was attributed to the sulphate groups of sugar chains such as ulvan. There was no change in the ratio of carbon, hydrogen, nitrogen, and sulfur in both light and dark periods throughout the cultivation. This suggests that the elemental composition of *U. meridionalis* is always constant regardless of the extensive accumulation of photosynthetic products during the light period. The carbon uptake by *U. meridionalis* was measured from the dry weight, and carbon content was obtained by CHNS analysis. The amount of carbon fixed per unit dry algae reached 16.8 mg C fixed g dry wt^−1^ h^−1^ (0–4 h), 15.7 mg C fixed g dry wt^−1^ h^−1^ (4–8 h), and 13.7 mg C fixed g dry wt^−1^ h^−1^ (8–12 h), which exceeded those of tubular and sheet-like *Ulva* sp. (5.16 mg C fixed g dry wt^−1^ h^−1^ on average) reported by Littler et al.^33^.

### Incorporation of ^13^C in NDP sugars

Subsequently, NDP sugar was extracted according to the method of Räbina et al.^34^, and the incorporation of ^13^C into the NDP sugar was measured by FT-ICR-MS. Low molecular weight compounds such as pigments were first extracted from *U. meridionalis* samples with 75% aqueous ethanol. The amount of ethanol extract used was consistent over time and was 37.7 ± 4.5% on average during both light and dark periods (Supplementary Fig. S2). 75% aqueous ethanol extract was dissolved in aqueous NaHCO_3,_ and the soluble fraction was applied to ENVIcarb solid-phase extraction (SPE) cartridge. This column is suitable for the separation of highly polar compounds. Highly polar NDP sugars were adsorbed into the column, and then the NDP sugar was eluted with 25% acetonitrile containing triethylammonium acetate buffer (TEAA; 50 mM, pH 7) to recover NDP sugars. The NDP sugar was analysed by FT-ICR-MS equipped with ESI (Bruker solariX). The MS spectra were recorded in negative-ion mode since the NDP sugars are negatively charged. Table 1 shows the carbon number, monoisotopic mass, and m/z of ^13^C-labelled UDP-pentose, UDP-deoxyhexose, UDP-hexose, and UDP-hexuronic acid as precursors of ulvan and starch. The monoisotopic mass is indicated as M_0_, and the mass changes from M_0_ to M_0+15_, depending on the carbon number of each NDP sugar (carbon numbers = 14 or 15) by ^13^C labelling, were analysed. For example, UDP-pentose with 14 carbons (M_0_ = 535), which corresponds to the precursor of Xyl, exhibits M_0+14_ when it is completely labelled with ^13^C. Similarly, UDP-hexose and UDP-hexuronic acid correspond to precursors of Glc and Gal and GlcA, respectively.

**Table 1:** Accurate mass analysis of nucleic diphosphate sugars by FT-ICR-MS.

NDP sugar	Corresponding monosaccharide(s)	Number of carbons	observed Monoisotopic mass (m_0_, *m/z*)	^13^C-labeled mass (*m*/*z*)
UDP-pentose	Xyl	14	535.05264	549.10112
UDP-deoxy-hexose	Rha	15	trace (549)	trace (564)
UDP-hexose	Glc, Gal	15	565.06293	580.11671
UDP-hexuronic acid	GlcA	15	579.04417	594.09604
UDP-*N*-acetyl-hexose	Not identified	17	606.09319	623.15260

**Figure 2:**
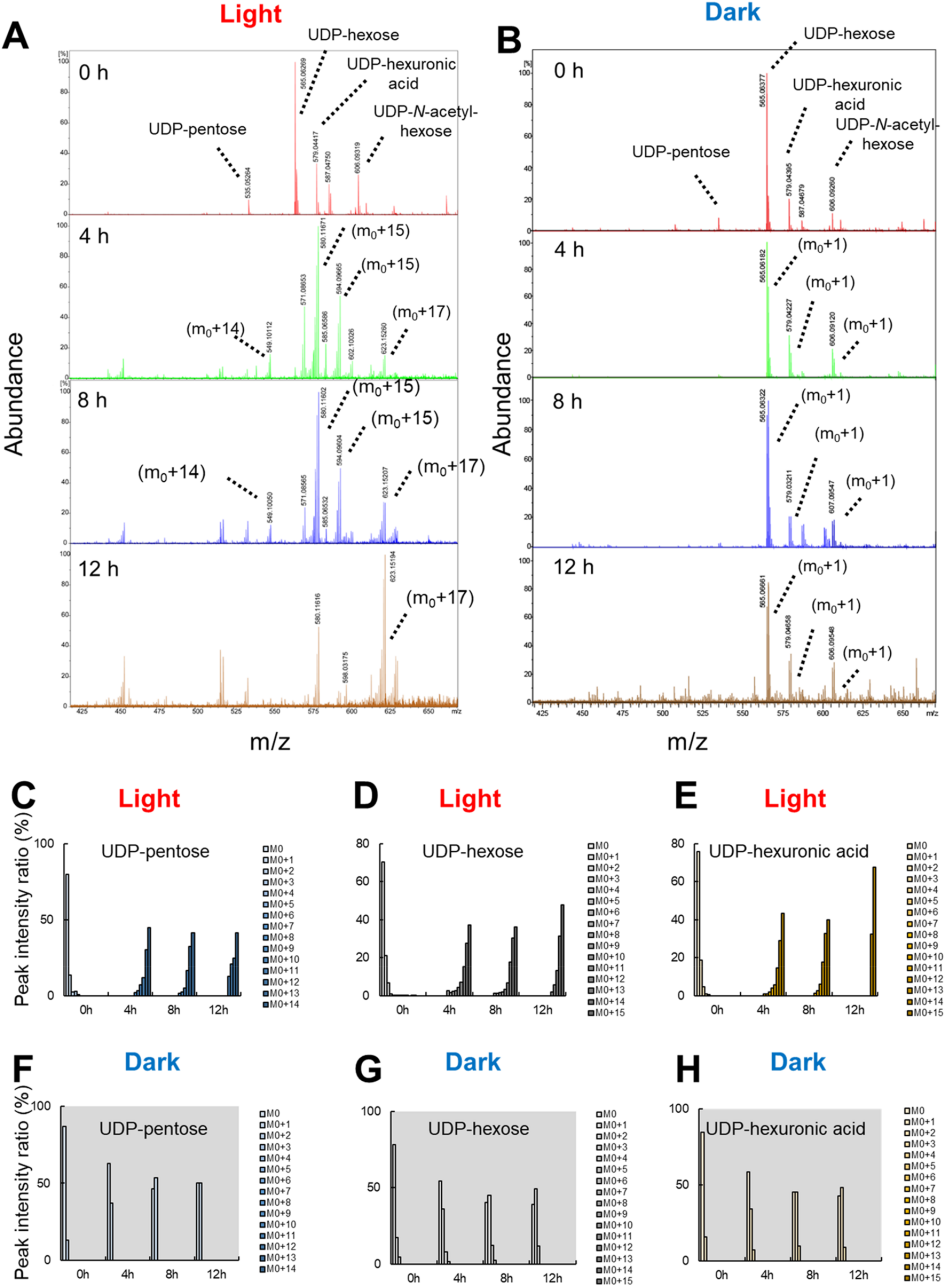
FT-ICR-MS analysis of ^13^C-incorporated nucleic diphosphate sugars produced under (**A**) light and (**B**) dark conditions. Comparisons of monoisotopic mass (M_0_) and ^13^C-labelled mass (M_0 + X_, X = 1–15) under (**C**–**E**) light and (**F**–**H**) dark.

Figure 2 shows the temporal change in the FT-ICR-MS spectrum of ^13^C-labelled NDP sugars. Peaks with m/z corresponding to UDP-pentose, UDP-hexose, and UDP-hexuronic acid were detected. On the other hand, UDP-deoxyhexose, which is considered to be a precursor of Rha, was not detected. Similarly, dTDP-Rha (M_0_ = 547), which is the precursor of Rha in microorganisms, was not detected. In addition, m/z corresponding to UDP-N-acetyl-hexose was detected in both light and dark periods. However, the corresponding monosaccharide to UDP-N-acetyl-hexose has not been identified. Since the ^13^C labelling may cause an overlap in M_0+x_ peaks of several compounds, FT-ICR-MS measurement of non-labelled NDP sugars was also performed using *U. meridionalis* cultured in a non-labelled medium under the same conditions (Supplementary Fig. S3). The monoisotopic mass peaks corresponding to above NDP sugars were detected; thus, their presence was confirmed.

In Fig. 2C–H, the changes in the mass of each NDP sugar are extracted, and they were expressed as the ratio of the individual masses to the sum of M_0_ to M_0+X_ (X = 14, 15). ^13^C was rapidly incorporated into NDP sugars under light conditions. After 4 h of ^13^C labelling, M_0+14_ (UDP-pentose) and M_0+15_ (UDP-hexose, UDP-hexuronic acid) were the major peaks followed by partially labelled peaks of M_0_ to M_0+14_. The ratio of ^13^C incorporated in UDP-hexose was constant at 4–8 h. After 12 h, the peak of M_0+15_ increased; however, M_0+15_ did not reach 100% even after 12 h. This suggested that *Ulva* also used non-labelled metabolites, which were accumulated before ^13^C labelling, for production of NDP sugars. With UDP-hexuronic acid, the peak of M_0+15_ at 12 h reached 70% of the total, suggesting that the UDP-GlcA was generated from ^13^C-labeled UDP-Glc. On the other hand, UDP-pentose showed lesser uptake of ^13^C than that of UDP-hexose and UDP-hexuronic acid. ^13^C incorporation into UDP-pentose was slow because the formation of UDP-Xyl is downstream of UDP-GlcA formation. During the dark period, ^13^C uptake was much lower than that in the light period, but ^13^C uptake still occurred to some extent. Peaks corresponding to M_0+1_ to M_0+2_ were observed and increased over time. Carbon fixation, also called dark reaction, proceeded even in the absence of light. However, carbon uptake was particularly low in the dark period.

### Incorporation of ^13^C in ulvan and starch

**Figure 3:**
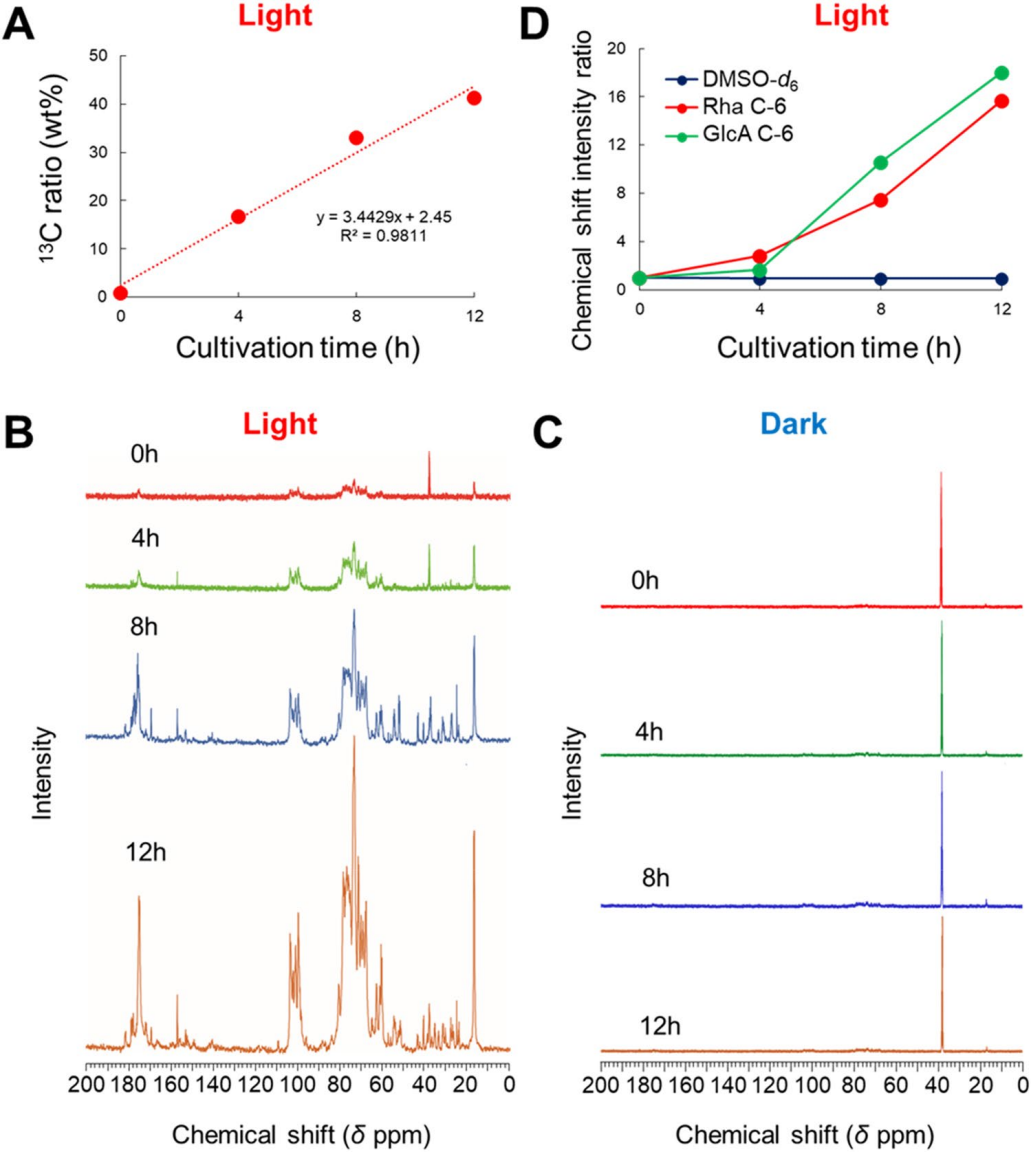
(**A**) The amount of incorporated ^13^C in the hot water extract as cultivation time increased. ^13^C-NMR spectra of ^13^C-labelled ulvan extracted from *Ulva meridionalis* cultivated under (**B**) light and (**C**) dark. (**D**) Temporal changes in the chemical shift intensities of Rha C-6 and GlcA C-6.

The transfer of ^13^C to matrix polysaccharides and starch of *U. meridionalis* was investigated using ^13^C-NMR. Polysaccharides were recovered through hot water extraction (90 °C, 1 h × 3) of residues after 75% aqueous ethanol extraction. The polymers in the hot water extract were recovered through ethanol precipitation. After freeze-drying, the incorporation of ^13^C into the hot water extract was evaluated by IR-MS. This fraction contained starch and ulvan but was not purified further because the amount of extract was small. Figure 3A shows the ^13^C ratio in the hot water extract. The incorporation rate of ^13^C into polysaccharides was the same as that for the whole algae body because their slopes were almost the same. Then, the hot water extract was dissolved in a mixed solvent of D_2_O-DMSO-*d*_6_ and analysed by ^13^C-NMR. Inverse-gated–decoupling provided the quantitative ^13^C spectra with ^1^H decoupling with a sufficient relaxation time of the *T*_1_^13^C^35^. However, in the present study, the repetition time was set to 10.69206 s since the *T*_1_^13^C was long^36^. DMSO-*d*_6_ contained in the solvent was used as an internal standard to determine the amount of ^13^C uptake into each carbon-containing ulvan.

Figure 3B,C shows the semiquantitative ^13^C-NMR spectra of the hot water extract during the light and dark periods. A clear chemical shift was observed in the light period, and the intensity increased with time. These chemical shifts were similar to those of *U. armoricana* ulvan because there were similar signals for G1, Gg1, R’1, and R1 at around 100 ppm^23^. Chemical shifts in the range of 60 ppm–100 ppm were attributed to C–OH of sugar moieties, but they were difficult to analyse in detail because they overlapped with each other. Therefore, the signals at 17 ppm due to C-6 of Rha and 176 ppm due to the carboxyl group of C-6 of GlcA were analysed because they were completely separate from the other signals. A normalised intensity against those of 0 h is summarised in Fig. 3D. The transfer of ^13^C to Rha and GlcA occurs later compared with the transfer of ^13^C to the hot water extract. The intensity did not change so much at 4 h but then increased from 8 h and finally reached about 16 times that of the initial intensity by 12 h. This suggests that the rate of Rha and GlcA incorporation into ulvan are almost the same. On the other hand, no clear signals other than that of DMSO-*d*_6_ were observed in the dark period.

### Incorporation of ^13^C in monosaccharides in ulvan and starch

**Figure 4:**
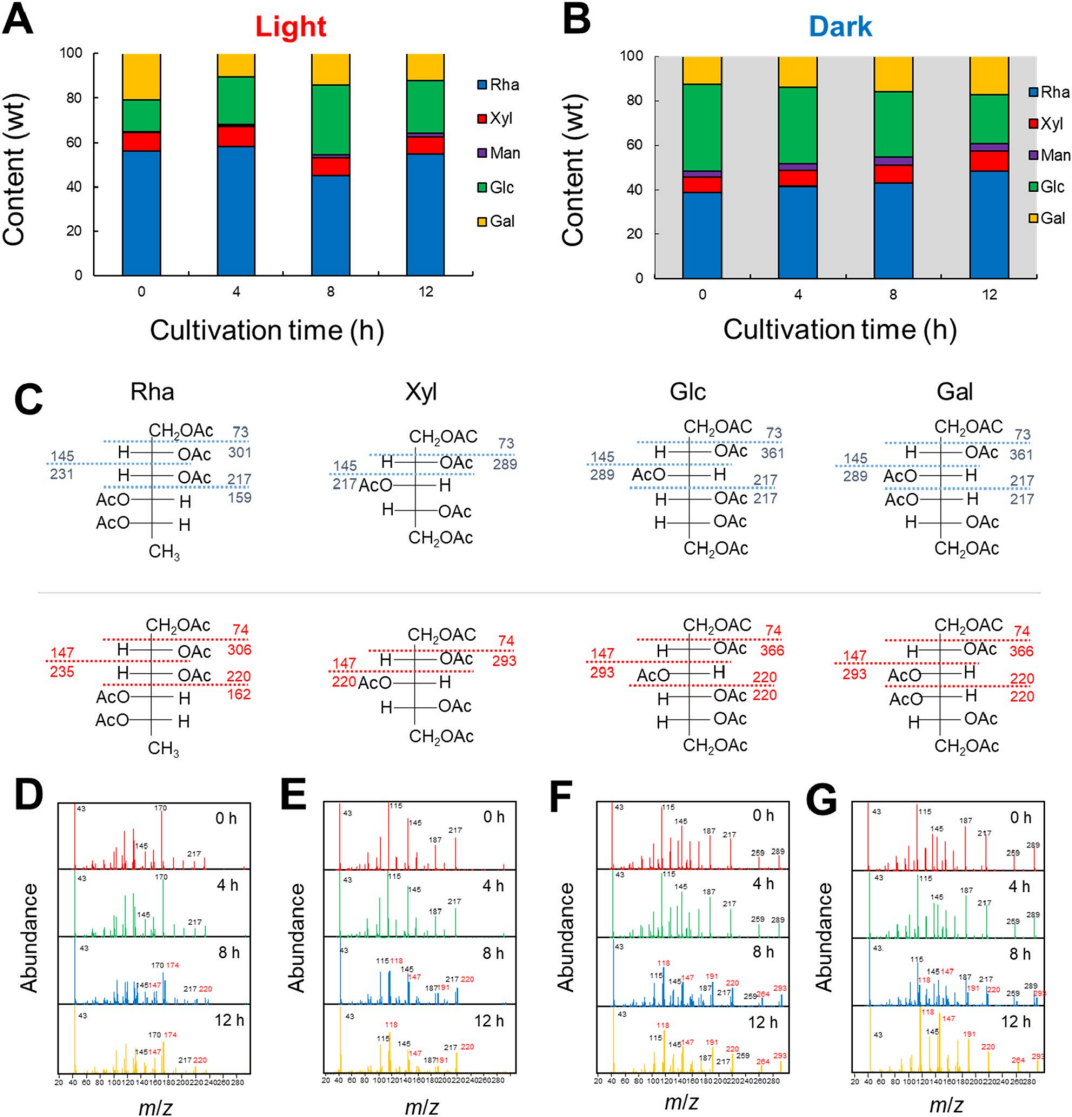
EI-MS spectra of alditol acetate derivatives of neutral sugars obtained by hydrolysis of ulvan extracted from *U. meridionalis* cultivated under light. Monosaccharide composition of hot-water extracts obtained under (**A**) light and (**B**) dark periods. (**C**) Fragmentation patterns of alditol acetate derivatives of neutral sugars with and without ^13^C-labelling. EI-MS spectra of (**D**) rhamnose, (**E**) xylose, (**F**) glucose, and (**G**) galactose.

Further, ^13^C incorporation at the monosaccharide level was analysed by GC–MS. Hot water extracts were hydrolysed with TFA (2 M), derivatised to alditol acetate, and then analysed by GC–MS. Figure 4A,B show the weight ratio of Rha, Xyl, Man, Glc, and Gal. Rha and Xyl were attributed to ulvan, while Glc was attributed to starch. Therefore, we focused on the ^13^C/^12^C ratios of Rha and Glc as representatives of ulvan and starch. The ratio of Rha was the highest in all conditions. On the other hand, Glc varied greatly depending on the cultivation time. In the light period, the Glc ratio increased from 0 to 8 h. This Glc increase was attributed to starch production during the light period. Then, the Rha ratio increased up to 12 h due to the deposition of ulvan and resulted in a relatively low Glc ratio. During the dark period, the Glc ratio gradually decreased with time. This is probably because the starch synthesised in the light period was consumed in the dark period. Therefore, the ratio of Rha and Xyl gradually increased as the ratio of Glc decreased.

The incorporation of ^13^C into each monosaccharide was investigated in detail using EI-MS spectra. Figure 4C shows changes in typical fragmentation patterns of neutral sugars due to the introduction of ^13^C. Replacement of the fragment ions with ^13^C changes m/z from + 1 to + 4. Figure 4D–G show temporal changes in the fragmentation patterns of each neutral sugar in the light period. The m/z changes following the incorporation of ^13^C are highlighted with bold lines and red letters. For example, in Rha, a fragment corresponding to m/z 217 began to migrate to 220 in 8 h. The peak intensity of 220 was 2.1 times higher than that of 217 at 12 h. The average ^13^C/^12^C ratio in Rha was about 1.3 after 12 h (Supplementary Table S2). On the other hand, the average ^13^C/^12^C ratio in Glc was 3.8 after 12 h, which was higher than that of Rha (Supplementary Table S2). This indicates that the vigorous starch biosynthesis in the light period increases the level of ^13^C incorporation into Glc. On the other hand, ulvan biosynthesis represented by the ^13^C/^12^C ratio of Rha was slower than starch biosynthesis.

## Discussion

This study performed ^13^C labelling cultivation of *U. meridionalis* to understand the mechanisms by which they grow up to fourfold a day. We tracked the fresh and dry weight changes, ^13^C uptake in whole algae, the formation of NDP sugars, matrix polysaccharides, and monosaccharides during the light and dark periods. Compared to the previous ^14^C labelling of *Ulva*^25^, enriched ^13^C was useful to track the carbon fixation into matrix and storage polysaccharides through NDP sugars by using IR-MS, FT-ICR-MS, ^13^C-NMR and GC–MS. In the light period, photosynthesis promotes strong carbon fixation. Carbon fixed in the Calvin cycle is converted into UDP-Glc via fructose 6-phosphate and then UDP-GlcA and UDP-Xyl. Labelling of these NDP sugars already occurred at an early stage after the start of the light period. Meanwhile, the transfer of ^13^C to matrix polysaccharides was delayed by the formation of NDP sugars and occurred more than 4 h after the start of the light period. Percival et al. studied carbon fixation in *U. lactuca* using radioactive ^14^C as a tracer and found that sucrose was the earliest product, detected 10 min after labelling, followed by starch formation^25^. Then, ulvan was formed after a delay of about 3 h. Therefore, *U. meridionalis* has a similar sugar biosynthesis system to that of *U. lactuca*. However, the carbon fixation speed by *U. meridionalis* further exceeded the average primary productivity by other thin tubular and sheet-like seaweeds (mainly *Ulva* seaweeds) by 2.65–3.26-fold^33^.

The change in fresh weight during growth did not show a large difference between the light and the dark periods (Fig. 1A,B). This was also confirmed by the growth behaviour when cultured in the unlabelled medium (Supplementary Fig. S5A). On the other hand, the change in dry weight differed greatly between the light and the dark periods. The dry weight increased clearly in the light period, but there was almost no change in the dark period. The similar behaviour was also observed with the unlabelled medium (Supplementary Fig. S5B). This indicates that most of the carbon fixation products are obtained by photosynthesis during the light period, which is completely consistent with the ^13^C uptake behaviour. Meanwhile, water content increased during the dark period. As a result, the fresh weight increased linearly in both light and dark periods.

Based on the above carbon uptake behaviours, we turned to prolong the light period to test if it is an effective method of accumulating more fixed carbon and promoting growth. Greater carbon fixation will be effective for biomass production and carbon dioxide fixation. Supplementary Fig. S4 shows the growth behaviour when the light:dark regime was varied from 12:12 to 24:0. Under a light:dark regime of 14:10, the daily growth rate increased, however, the growth rate did not increase when the light period was prolonged above 14 h. This suggests that the dark period is also important for *U. meridionalis* growth. According to Hiraoka et al., cell division should occur at least twice a day to grow fourfold a day^6^. According to Kuwano et al., the cell division cycle of *U. partita* (= *Enteromorpha compressa* in Kuwano et al.) is controlled by the diurnal cycle, and a dark period is required for 6 h^37^. During the dark period, the number of cells increase by cell division. That is, cell division occurs using photosynthetic metabolites produced under the light period. In fact, the percentage of Glc in the hot water extract-hydrolysate reduced in the dark period. This indicates that starch is consumed as an energy source for cell division. In addition, McArthur et al. reported that cell wall deposition occurs at the end of cytokinesis through light and electron microscopy of *U. intestinalis* during mitosis^38^. That is, the ulvan obtained during the light period also contributes to the formation of the cell wall, accompanying cell division during the dark period.

**Figure 5:**
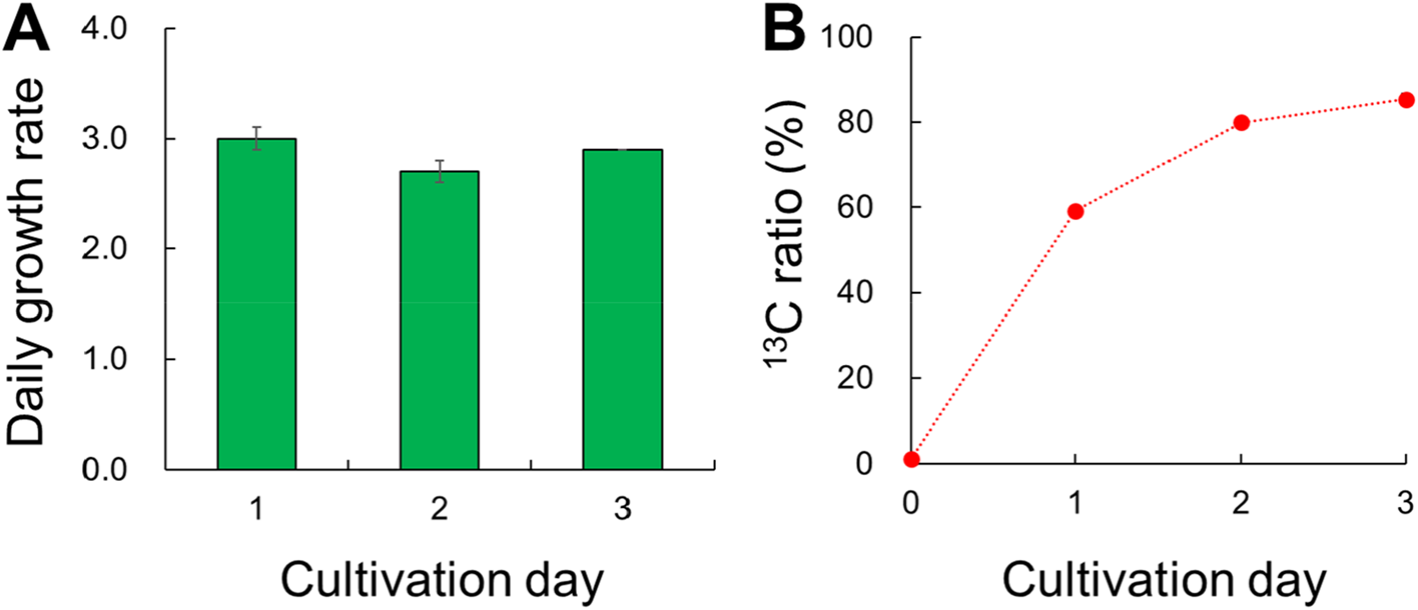
The ^13^C concentrating cultivation of *Ulva meridionalis*. (**A**) Daily growth rate and (**B**) ^13^C ratio.

The optimal light:dark regime of *U. meridionalis* was the long-day condition of around 14:10. This is consistent with the fact that *U. meridionalis* was first discovered in summer and grows at high water temperatures^7^. Then, we examined how much carbon could be fixed by *U. meridionalis* using ^13^C labelling under the light:dark regime of 14:10. A closed flask bubbling with N_2_ gas was used to enable long-term ^13^C labelling (Supplementary Fig. S6). The same ^13^C-labelled EASW mentioned above was added to the flask and filled with nitrogen gas so as not to contact the ^12^C in the atmosphere. Nitrogen gas was circulated using a diaphragm pump to appropriately agitate the algal bodies. The ^13^C medium was replaced every 24 h so that the medium was always fresh. Under these conditions, *U. meridionalis* grew three times each day (Fig. 5A). Uptake of ^13^C at 72 h reached approximately 85%. This confirms the very high carbon fixation capacity of *U. meridionalis* (Fig. 5B).

In conclusion, the high growth rate of *U. meridionalis* is associated with its strong carbon fixation ability. Through ^13^C labelling in the light period, the NDP sugar was rapidly labelled as the precursor of polysaccharides; then, ^13^C was transferred to ulvan later. In contrast, carbon uptake hardly occurred in the dark period. Cell division was carried out in the dark period using the carbon fixed during the light period. For instance, the accumulated starch may be used as an energy source for cell division. Further, ulvan may be used for cell wall formation at the end of cell division. Under optimal long-day conditions, 90% of ^13^C labelling was achieved within 72 h. We conclude that *U. meridionalis* exhibits a high carbon fixation capacity that contributes to its high growth potential.

## Methods

### Pre-cultivation of *U. meridionalis*

The pre-cultivation of *U*. *meridionalis* was conducted according to the procedure of Hiraoka et al.^6^. Namely, *U*. *meridionalis* stock was transferred to non-^13^C labelled EASW (Supplementary Table S1) for 8 days^30–32^. The light irradiation was operated at 50 μmol m^−2^ s^−1^ under a light:dark regime of 12:12. The EASW medium was exchanged every second day. The flask size was gradually increased from 1 L (4 days) to 2 L (2 days) and 5 L (2 days) depending on the growth of *U. meridionalis* during pre-cultivation (Supplementary Fig. S7). All cultivation were performed at 25 °C. The *U. meridionalis* were mixed using a stirring bar.

### ^13^C labelling of *U. meridionalis*

**Figure 6:**
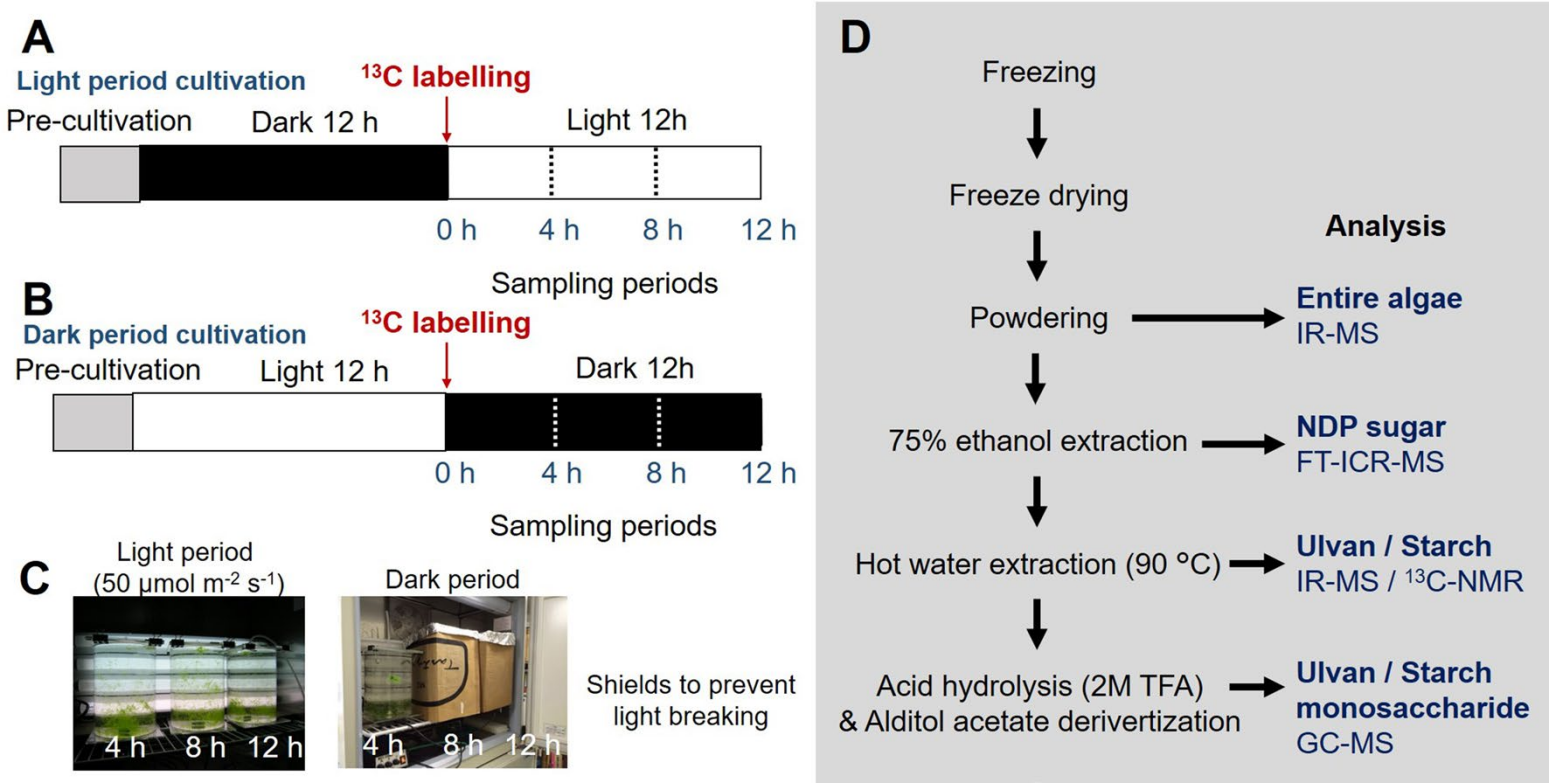
The ^13^C labelling and sampling periods of *Ulva meridionalis* under (**A**) light and (**B**) dark. (**C**) The photographs of each cultivation tank for sampling *U. meridionalis* after 4–12 h. (**D**) Fractionations and analyses of nucleic diphosphate sugar, ulvan / starch, and ulvan / starch monosaccharides.

Figure 6A,B show the schedule for ^13^C-labelling cultivation and sampling of *U. meridionalis*. First, *U. meridionalis,* in a vegetative growth stage, was pre-cultured using non-labelled EASW under a 12:12 light:dark regime. *U. meridionalis* was transferred into a ^13^C-labelled EASW medium containing ^13^C-NaHCO_3_ (99%, Cambridge Isotope Laboratories Inc., MA, USA). as the sole carbon source (Supplementary Table S1). ^13^C-labelled *U. meridionalis* was collected every 4 h under light (50 μmol m^−2^ s^−1^) and dark periods (Fig. 6A–C). The medium was homogeneously mixed by a stirring bar. The apparent amount of O_2_ generation was monitored from the amount of the gas emitted from the cultivation tank. After collecting the ^13^C-labelled *U. meridionalis*, their fresh weight was measured, then immediately frozen, lyophilised, and powdered to measure the dry weight (Fig. 6D). The total amount of incorporated ^13^C was measured by IR-MS (RMI-2, Hitachi, Ltd., Tokyo, Japan). The approximate elemental analysis was performed using the CHNS analyser (FlashEA 1112; Thermo Fisher Scientific Inc., MA, USA). Dried samples were further used for NDP sugar and polysaccharide analyses (Fig. 6D).

### NDP sugar analysis by FT-ICR-MS

NDP sugars were extracted and purified by SPE according to the methods of Räbina et al. with slight modifications^34^. Namely, the dried powders of *U*. *meridionalis* were extracted three times with 75% aqueous ethanol, and then the extracts were dried using an evaporator. The dried extracts were suspended in aqueous NaHCO_3_ (10 mM) solution. The solubilised fractions were loaded on the ENVI-Carb SPE cartridge (Sigma-Aldrich Co. LLC., MO, USA). The SPE cartridge was successively washed with ultra-pure water, TEAA buffer (50 mM, pH 7, Wako Pure Chemical Industries Ltd., Kyoto, Japan) solution, and 25% aqueous acetonitrile solution. The cartridge was finally eluted with 25% aqueous acetonitrile containing TEAA. The extracted NDP-sugars were analysed by the Bruker solariX FT-ICR-MS (Bruker Co., MA, USA) operated with ESI in negative ion mode with 20 μL min^−1^ of sample infusion.

### Ulvan analysis by semiquantitative ^13^C-NMR and GC–MS

Ulvan was extracted from the residue following aqueous ethanol (75%) extraction with hot water (90 °C, 1 h × 3). Ulvan was recovered by ethanol precipitation and then freeze-dried. The ^13^C uptake in ulvan was measured by IR-MS. Semiquantitative ^13^C-NMR of ^13^C-labelled ulvan was performed by the inverse-gated decoupling method using JEOL NMR ECA600 (150 MHz)^35,36^. Ulvan was re-solubilised in D_2_O-DMSO-*d*_6_ (92:8) mixed solvent. NMR spectra were recorded at 40 °C with a repetition time of 10.69206 s. The chemical shift intensities were normalised to the DMSO-*d*_6_ internal standard. The monosaccharide composition of ulvan was analysed by GC–MS (Agilent GC 8690 N and MS 5973; Agilent Technologies, CA, USA). Ulvan was hydrolysed into monosaccharides by aqueous trifluoro acetate (TFA; 2 M, 121 °C, 1 h). After removing TFA, the monosaccharides were derivatised to alditol acetate and analysed by GC–MS with a column of CBP-1 (Shimadzu GLC Ltd., Kyoto, Japan) operated at 140–160 °C (2 °C min^−1^).

## Supplementary information

Supplementary Information.

## Data Availability

All relevant data are included in the main text and Supplementary Information.
