# Supplementary material for: Probing rapid carbon fixation in fast-growing seaweed Ulva meridionalis using stable isotope 13C-labelling
Source: Sci Rep. 2020 Nov 23;10:20399. doi: 10.1038/s41598-020-77237-1 (PMC7684289; doi:10.1038/s41598-020-77237-1)
Supplement: Supplementary file 1 — Supplementary Information. [file 41598_2020_77237_MOESM1_ESM.docx]

**Supplementary information**

**Probing rapid carbon fixation in fast-growing seaweed *Ulva meridionalis* using stable isotope ^13^C-labelling**

Shuntaro Tsubaki ^a*^, Hiroshi Nishimura ^b^, Tomoya Imai ^b^, Ayumu Onda^c^, Masanori Hiraoka ^d^

^a^ School of Materials and Chemical Technology, Tokyo Institute of Technology, E4-3, 2-12-1, Ookayama, Meguro-ku, Tokyo 152-8552, Japan

^b^ Research Institute for Sustainable Humanosphere, Kyoto University, Gokasho, Uji 611-0011, Japan.

^c^ Research Laboratory of Hydrothermal Chemistry, Faculty of Science, Kochi University, 2-17-47 Asakurahonmachi, Kochi 780-8073, Japan.

^d^ Usa Marine Biological Institute, Kochi University, Inoshiri, Usa, Tosa, Kochi 781-1164, Japan.

*Corresponding author;

Tel +81-3-5734-3735

E-mail; shuntaro.tsubaki@gmail.com, tsubaki.s.aa@m.titech.ac.jp

**Supplementary Table 1.** Chemical composition of the enriched artificial seawater (EASW) with a sole ^13^C source

| **Compound** | **Content**  **(g/L)** | **Compound** | **Content**  **(g/L)** | **Compound** | **Content**  **(g/L)** |
| --- | --- | --- | --- | --- | --- |
| NaCl | 21.2 | SrCl_2_・6H_2_O | 0.0219 | CoSO_4_・7H_2_O | 0.00016 |
| Na_2_SO_4_ | 3.55 | NaNO_3_ | 0.0467 | Na_2_MoO_4_ | 0.00013 |
| KCl | 0.599 | NaH_2_PO_4_・2H_2_O | 0.00328 | Na_2_SeO_3_ | 0.000017 |
| ^13^C-NaHCO_3_***** | 0.174 | Na_2_SiO_3_・9H_2_O | 0.0298 | NiCl_2_・6H_2_O | 0.00015 |
| KBr | 0.0863 | FeCl_3_・6H_2_O | 0.00177 | Thiamine-HCl | 0.0001 |
| H_3_BO_3_ | 0.023 | Na_2_EDTA・2H_2_O | 0.00244 | Biotin | 0.0002 |
| NaF | 0.0028 | ZnSO_4_・7H_2_O | 0.000073 | Vitamin B_12_ | 0.0001 |
| MgCl_2_・6H_2_O | 8.38 | MnSO_4_・5H_2_O | 0.000583 |  |  |
| CaCl_2_・2H_2_O | 1.34 | Na_2_・EDTA・2H_2_O | 0.00309 |  |  |

* ^13^C-labelled NaHCO_3_ (99 %) Cambridge Isotope Laboratories

**Supplementary Table 2.** The average ratio of EI-MS fragments

| **Monosaccharides** | **Ratio of ^13^C incorporated fragment ion (%)** | | **Fragment ions (m/z)** |
| --- | --- | --- | --- |
|  | **Light 8 h** | **Light 12 h** |  |
| Rha | 42.7 ± 9.9 | 57.2 ± 14.7 | 145, 170, 217 |
| Xly | 49.6 ± 6.0 | 58.0 ± 17.0 | 115, 145, 187, 217 |
| Glc | 60.5 ± 12.3 | 78.8 ± 12.3 | 115, 145, 187, 217, 259, 289 |
| Gal | 32.6 ± 3.9 | 93.6 ± 15.5 | 115, 145, 187, 217, 259, 289 |

**
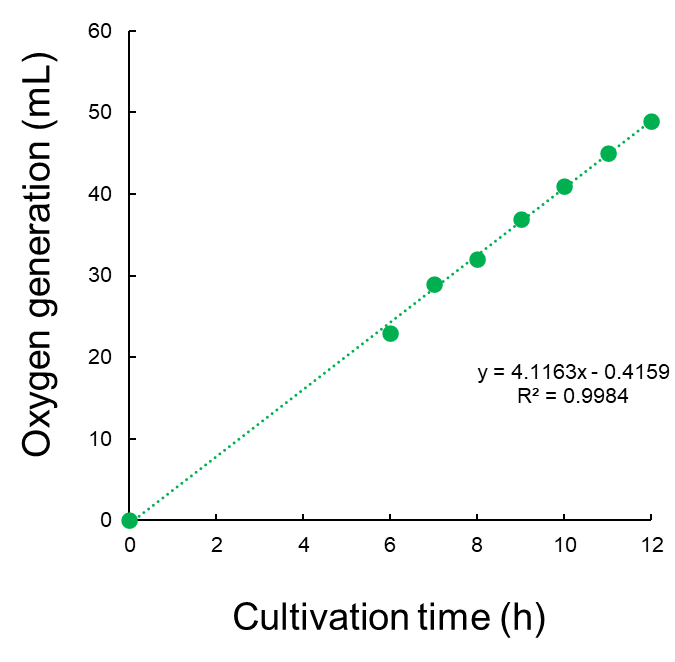
**

**Supplementary Figure 1.** Amount of oxygen generation under light condition

**
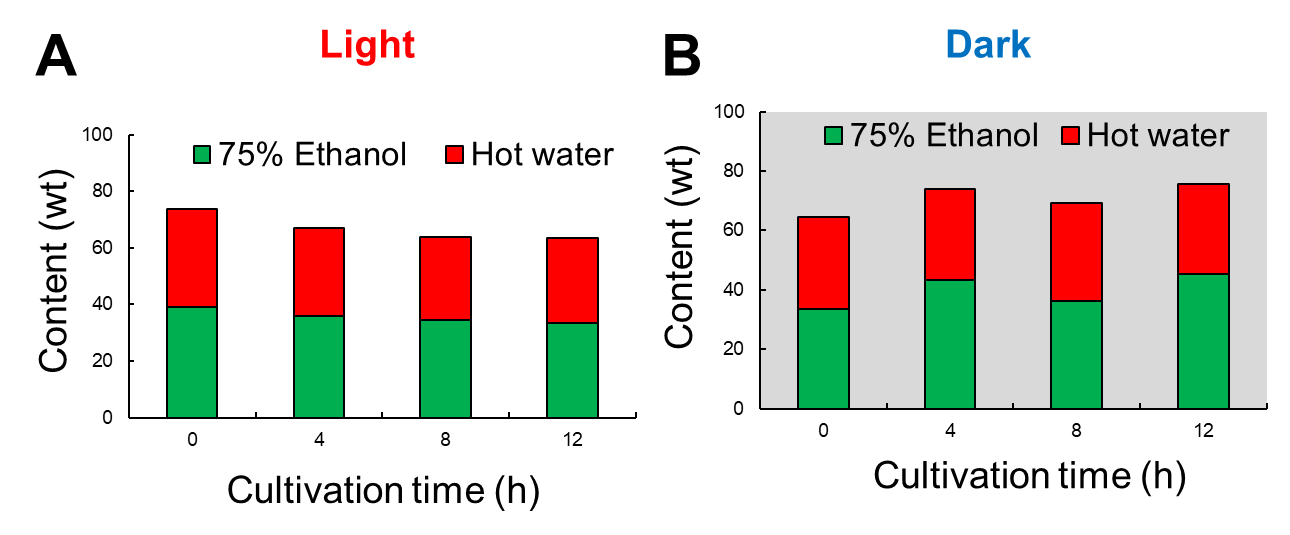
**

**Supplementary Figure 2.** Amounts of 75% aqueous ethanol and hot water extracts under **A** light and **B** dark.


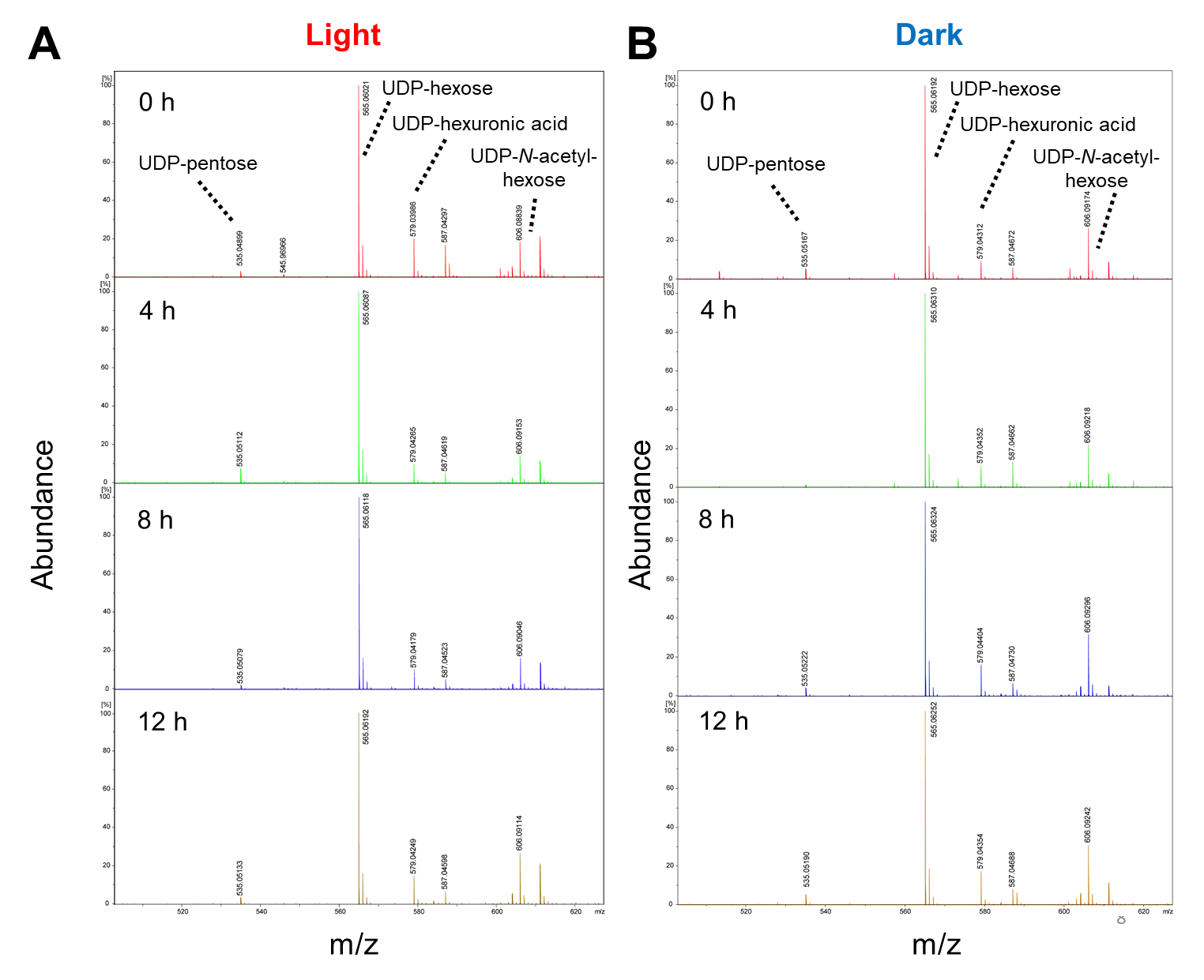


**Supplementary Figure 3.** FT-ICR-MS analysis of non-labelled nucleic diphosphate sugars produced under **A** light and **B** dark


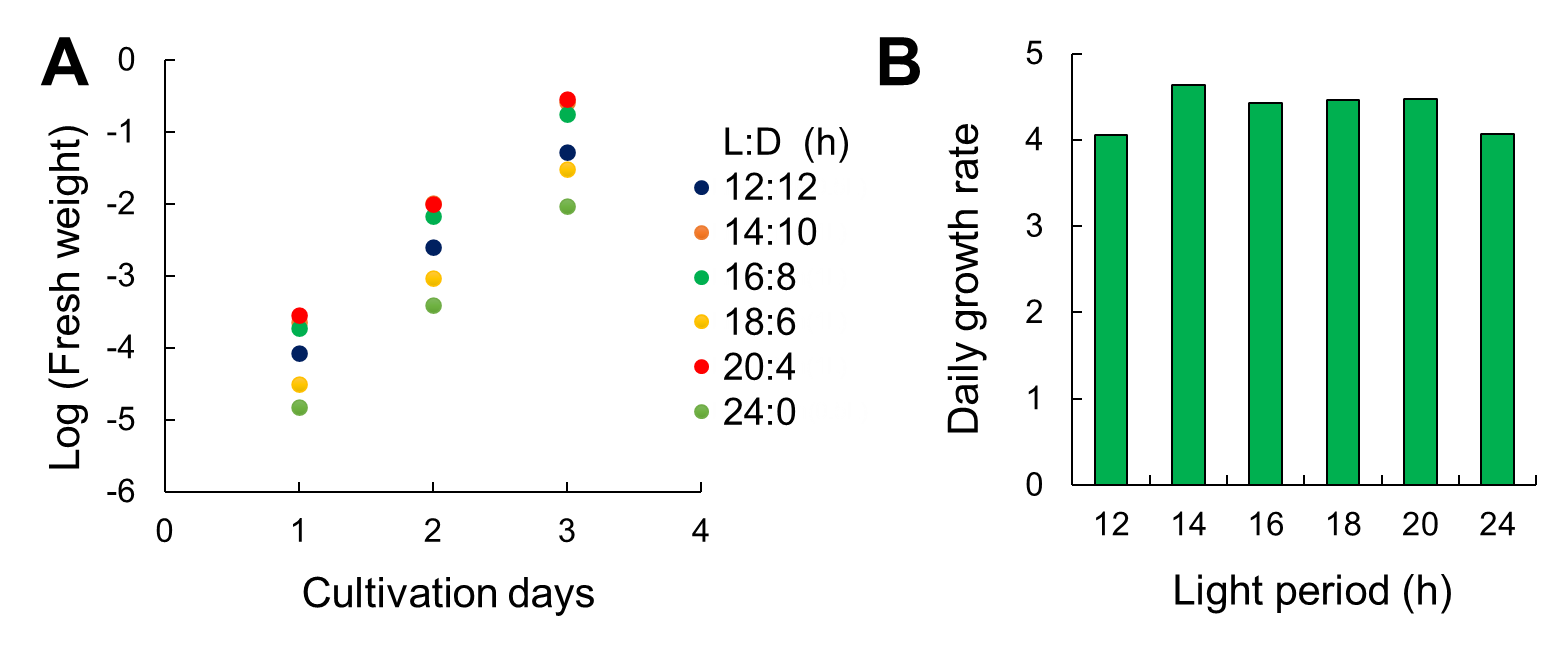


**Supplementary Figure 4.** The growth of *Ulva meridionalis* under long-day conditions. **A** Logarithm of fresh weight and **B** the corresponding daily growth rate.


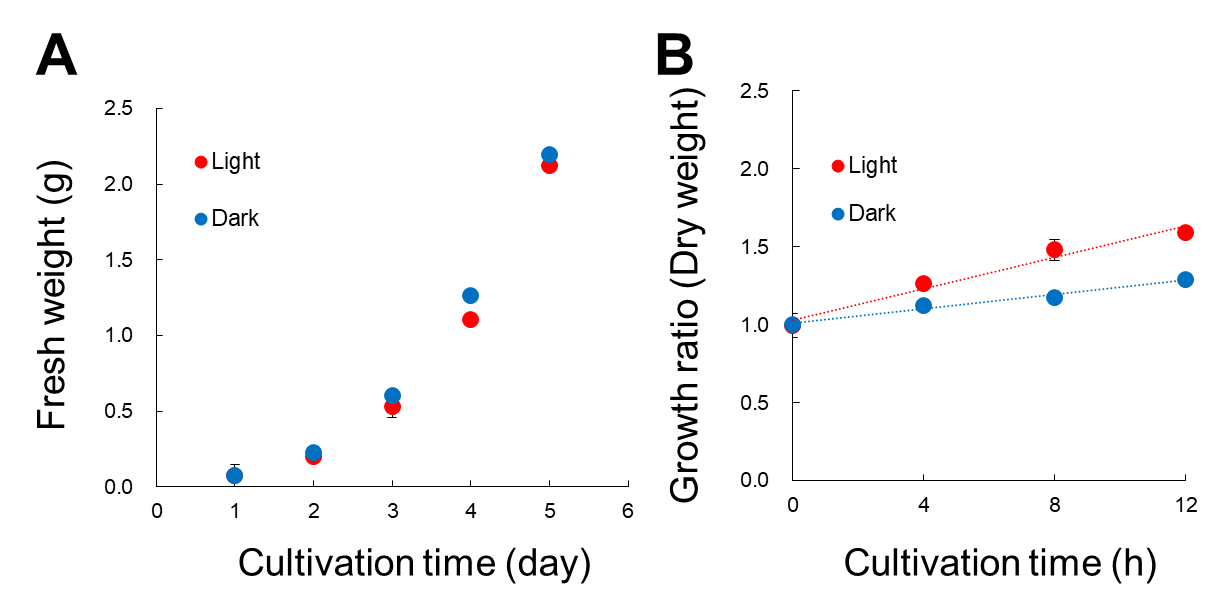


**Supplementary Figure 5 A** Fresh weight measured in light and dark conditions. **B** Growth ratio in dry weight under light and dark

**
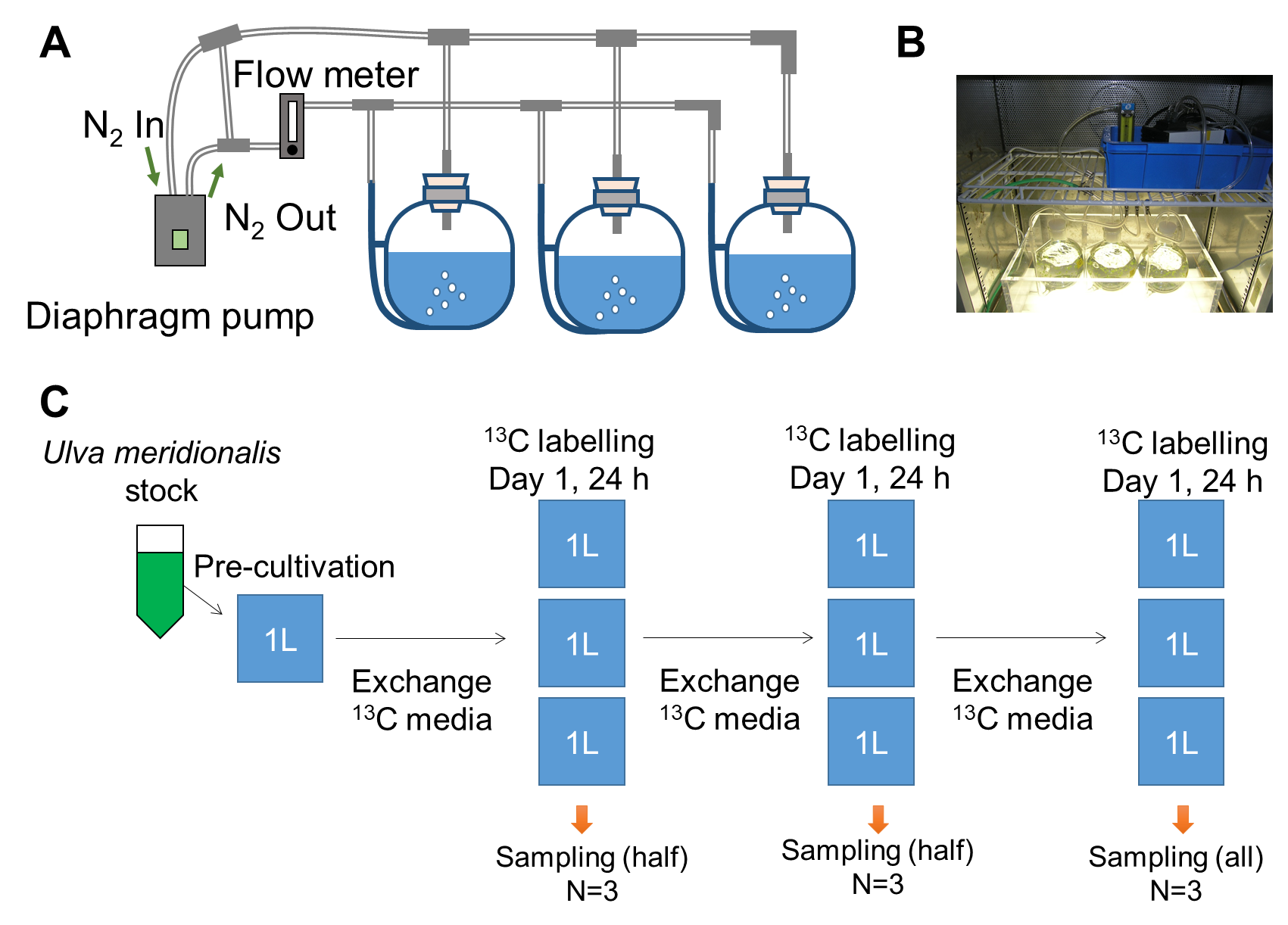
**

**Supplementary Figure 6.** **A** Set-up and **B** photograph for ^13^C concentrating cultivation of *Ulva meridionalis*. **C** Procedure for ^13^C labelling test for 72 h.


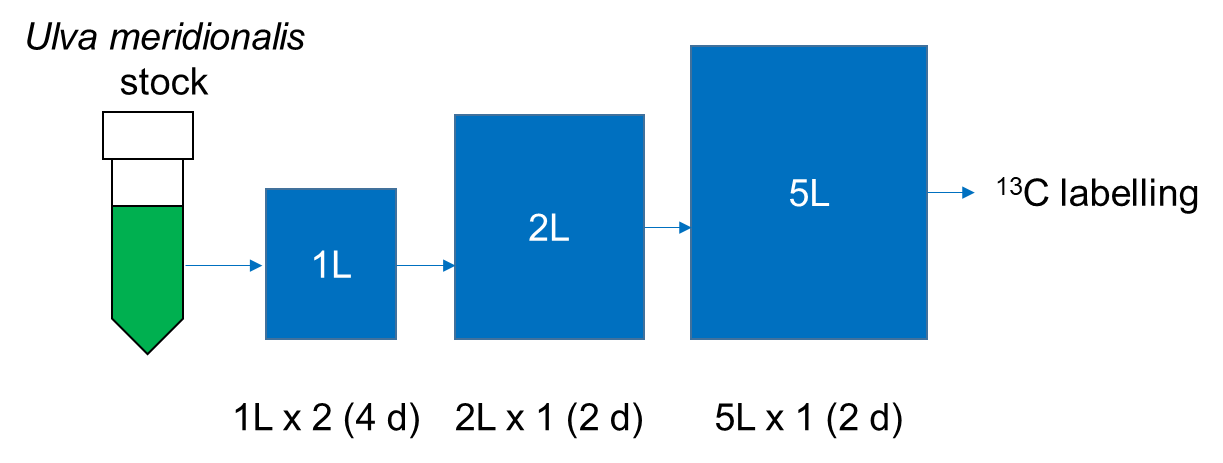


**Supplementary Figure 7.** Pre-cultivation procedure of *Ulva meridionalis* before ^13^C-labelling.
